# Frizzled-9+ Supporting Cells Are Progenitors for the Generation of Hair Cells in the Postnatal Mouse Cochlea

**DOI:** 10.3389/fnmol.2019.00184

**Published:** 2019-07-31

**Authors:** Shasha Zhang, Dingding Liu, Ying Dong, Zhong Zhang, Yuan Zhang, Han Zhou, Lingna Guo, Jieyu Qi, Ruiying Qiang, Mingliang Tang, Xia Gao, Chunjie Zhao, Xiaoyun Chen, Xiaoyun Qian, Renjie Chai

**Affiliations:** ^1^Key Laboratory for Developmental Genes and Human Disease, Ministry of Education, Institute of Life Sciences, Southeast University, Nanjing, China; ^2^Co-Innovation Center of Neuroregeneration, Nantong University, Nantong, China; ^3^Jiangsu Provincial Key Medical Discipline (Laboratory), Department of Otolaryngology—Head and Neck Surgery, Affiliated Drum Tower Hospital of Nanjing University Medical School, Nanjing, China; ^4^Department of Otolaryngology—Head and Neck Surgery, First Affiliated Hospital of Wenzhou Medical University, Wenzhou, China; ^5^Institute for Stem Cell and Regeneration, Chinese Academy of Science, Beijing, China; ^6^Jiangsu Province High-Tech Key Laboratory for Bio-Medical Research, Southeast University, Nanjing, China; ^7^Beijing Key Laboratory of Neural Regeneration and Repair, Capital Medical University, Beijing, China

**Keywords:** Frizzled-9 (Fzd9), cochlea, hair cell progenitor, hair cell generation, self-renew

## Abstract

Lgr5+ cochlear supporting cells (SCs) have been reported to be hair cell (HC) progenitor cells that have the ability to regenerate HCs in the neonatal mouse cochlea, and these cells are regulated by Wnt signaling. Frizzled-9 (Fzd9), one of the Wnt receptors, has been reported to be used to mark neuronal stem cells in the brain together with other markers and mesenchymal stem cells from human placenta and bone marrow. Here we used Fzd9-CreER mice to lineage label and trace Fzd9+ cells in the postnatal cochlea in order to investigate the progenitor characteristic of Fzd9+ cells. Lineage labeling showed that inner phalangeal cells (IPhCs), inner border cells (IBCs), and third-row Deiters’ cells (DCs) were Fzd9+ cells, but not inner pillar cells (IPCs) or greater epithelial ridge (GER) cells at postnatal day (P)3, which suggests that Fzd9+ cells are a much smaller cell population than Lgr5+ progenitors. The expression of Fzd9 progressively decreased and was too low to allow lineage tracing after P14. Lineage tracing for 6 days *in vivo* showed that Fzd9+ cells could also generate similar numbers of new HCs compared to Lgr5+ progenitors. A sphere-forming assay showed that Fzd9+ cells could form spheres after sorting by flow cytometry, and when we compared the isolated Fzd9+ cells and Lgr5+ progenitors there were no significant differences in sphere number or sphere diameter. In a differentiation assay, the same number of Fzd9+ cells could produce similar amounts of Myo7a+ cells compared to Lgr5+ progenitors after 10 days of differentiation. All these data suggest that the Fzd9+ cells have a similar capacity for proliferation, differentiation, and HC generation as Lgr5+ progenitors and that Fzd9 can be used as a more restricted marker of HC progenitors.

## Introduction

Sensorineural hearing loss is mainly caused by hair cell (HC) loss and is one of the most common health problems around the world. HC loss is irreversible in adult mammals, whereas HCs can be regenerated from supporting cells (SCs) in the inner ear of birds and fish (Rubel et al., 2013). Recent studies have shown that HCs can also be regenerated in newborn mice (Chai et al., 2012; Shi et al., 2012; Bramhall et al., 2014; Wang et al., 2015; Li et al., 2016; You et al., 2018; Zhang et al., 2018). However, this regenerative ability is quickly lost as the mice age (Bermingham-McDonogh and Reh, 2011; Chai et al., 2012; Shi et al., 2012; Bramhall et al., 2014; Cox et al., 2014).

Several signaling pathways have been reported to play important roles in HC regeneration. The up-regulation of canonical Wnt signaling induces the proliferation of sensory precursors in the postnatal mouse cochlea (Chai et al., 2012; Shi et al., 2012; Ni et al., 2016; Waqas et al., 2016b; Wu et al., 2016; Lu et al., 2017), while Notch inhibition induces mitotically generated HCs in the mammalian cochlea *via* activation of the Wnt pathway (Li et al., 2015; Ni et al., 2016; Waqas et al., 2016b; Wu et al., 2016). Previously, *Lgr5*, a Wnt target gene, has been reported to mark the progenitors of the inner ear, and Lgr5+ progenitors have the ability to regenerate HCs in the neonatal mouse cochlea (Chai et al., 2011, 2012; Shi et al., 2012; Waqas et al., 2016a; Zhang et al., 2017). Lgr5 is expressed in third-row Deiters’ cells (DCs), inner pillar cells (IPCs), inner phalangeal cells (IPhCs), and part of the lateral greater epithelial ridge (GER) cells, which is a large cell population and contains many different cell types (Chai et al., 2011, 2012; Shi et al., 2012).

Wnt signaling plays important roles in regulating HC progenitors and HC regeneration (Chai et al., 2012; Li et al., 2015; Liu et al., 2016; Ni et al., 2016; Waqas et al., 2016b; Lu et al., 2017). There are 10 Frizzled (Fzd) receptors (Fzd1-10), which are unconventional G-protein coupled receptors. Fzds contain a conserved cysteine-rich domain to which Wnt ligands bind with high affinity (MacDonald and He, 2012; Dijksterhuis et al., 2014).

Frizzled-9 (Fzd9) plays crucial roles in brain development, neuromuscular junction assembly, new bone formation, and tumor suppression (Winn et al., 2005; Zhao et al., 2005; Albers et al., 2011; Avasarala et al., 2013b; Heilmann et al., 2013; Aviles et al., 2014; Ramírez et al., 2016). In the brain, Fzd9 is selectively expressed in both the developing and adult hippocampus and is used to mark neural stem cells together with other markers (Van Raay et al., 2001; Zhao and Pleasure, 2004, 2005; Pollard et al., 2008; Trubiani et al., 2010; Zhou et al., 2010; Pedersen et al., 2012; Tian et al., 2012). In human bone marrow and chorionic placenta-derived mesenchymal stem cells, Fzd9 is also expressed on the cell surface and is used as a marker to isolate these cells (Battula et al., 2007, 2008; Buhring et al., 2007; Trubiani et al., 2010; Tran et al., 2011). Thus, we speculated that Fzd9 might also be expressed in the HC progenitors of the cochlea and thus might also be used as a HC progenitor marker in the inner ear.

Here, we used Fzd9-CreER/Rosa26-tdTomato mice to lineage trace the Fzd9+ cells in the postnatal cochlea and to investigate their HC generation ability compared to the Lgr5+ progenitors. Lineage tracing data showed that Fzd9 was expressed in IPhCs, inner border cells (IBCs), and the third-row DCs, but not in IPCs or GER cells at postnatal day (P) 3. Lineage tracing data showed that, similar to Lgr5+ progenitors, Fzd9+ cells also had HC generation ability after 6 days of lineage tracing *in vivo*. When isolated by flow cytometry and then cultured *in vitro*, Fzd9+ cells and Lgr5+ progenitors could form similar numbers of spheres in an *in vitro* sphere-forming assay. In a differentiation assay, the same number of Fzd9+ cells could generate a similar amount of HCs compared to Lgr5+ progenitors after 10 days of differentiation. Our work provides a new marker for HC progenitors and expands our knowledge of progenitor cell types in the inner ear.

## Materials and Methods

### Animals

Lgr5-EGFP-IRES-creERT2 mice (Stock #008875, Jackson Laboratory) and Rosa26-tdTomato reporter mice (Stock #007914, Jackson Laboratory) of both sexes were used in the experiments (Madisen et al., 2010). The Fzd9-CreER mice were a gift from Prof Chunjie Zhao from Southeast University (Zhou et al., 2010). We performed all animal procedures according to protocols that were approved by the Animal Care and Use Committee of Southeast University and that were consistent with the National Institute of Health’s Guide for the Care and Use of Laboratory Animals. We made all efforts to minimize the number of animals used and to prevent their suffering.

### Genotyping PCR

Transgenic mice were genotyped using genomic DNA from tail tips by adding 180 μl 50 mM NaOH, incubating at 98°C for 1 h, and adding 20 μl 1 M Tris-HCl pH 7.0. The genotyping primers were as follows: *Lgr5*: (F) 5′-CTG CTC TCT GCT CCC AGT CT-3′; wild-type (R) 5′-ATA CCC CAT CCC TTT TGA GC-3′; mutant (R) 5′-GAA CTT CAG GGT CAG CTT GC-3′. *tdTomato*: wild-type (F) 5′-AAG GGA GCT GCA GTG GAG T-3′; wild-type (R) 5′-CCG AAA ATC TGT GGG AAG TC-3′; mutant (F) 5′-GGC ATT AAA GCA GCG TAT C-3′; mutant (R) 5′-CTG TTC CTG TAC GGC ATG G-3′. *Fzd9*: (F) 5′-CAT ACC TGG AAA ATG CTT CTG TCC-3′; (R) 5′-ATT GCT GTC ACT TGG TCG TGG C-3′.

### *In vivo* Labeling and Lineage Tracing of Fzd9+ Cells in the Cochlea

Fzd9^CreER/+^ mice and Lgr5-EGFP^CreER/+^ mice were crossed with Rosa26-tdTomato mice separately to label and lineage trace Fzd9+ and Lgr5+ cells in the cochlea. To activate cre, Fzd9^CreER/+^Rosa26-tdTomato and Lgr5-EGFP^CreER/+^Rosa26-tdTomato double-positive mice were intraperitoneally (I.P.) injected with tamoxifen (4 mg/25 g body weight, Sigma) at P3, P7, or P14. Mice were killed at different time points, and the cochleae were examined.

### Immunostaining and Image Acquisition

Cochleae were fixed in 4% (w/v) paraformaldehyde for 24 h at room temperature and washed with PBS, and the cochleae from P7 and older mice were decalcified with 0.5 M EDTA for 1–3 days. The cochleae were then washed with PBS, dissected in HBSS, and blocked with blocking solution [5% (v/v) donkey serum, 0.5% (v/v) Triton X-100, 0.02% (w/v) sodium azide, and 1% (v/v) bovine serum albumin in PBS (pH 7.4)] for 1 h at room temperature and then incubated with primary antibodies diluted in PBT1 [2.5% (v/v) donkey serum, 0.1% (v/v) Triton X-100, 0.02% (w/v) sodium azide, and 1% (v/v) bovine serum albumin in PBS (pH 7.4)] at 4°C overnight. The cochleae were then washed with 0.1% (v/v) Triton X-100 in PBS (pH 7.4) three times and incubated with fluorescence-conjugated secondary antibody (Invitrogen), both diluted 1:400 in PBT2 [0.1% (v/v) Triton X-100 and 1% (v/v) bovine serum albumin in PBS (pH 7.4)] for 1 h at room temperature. The cochleae were mounted in anti-fade fluorescence mounting medium (DAKO) after washing three times with 0.1% (v/v) Triton X-100 in PBS (pH 7.4). The primary antibodies were anti-Myosin7a (Proteus Bioscience, #25-6790, 1:1,000 dilution in PBT1) and anti-Sox2 (Santa Cruz Biotechnology, #17320, 1:400 dilution in PBT1). A Zeiss LSM 710 confocal microscope was used to obtain the fluorescence images.

### Cryosections

Isolated cochleae were fixed in 4% (w/v) paraformaldehyde in PBS (pH 7.4) at room temperature for 4 h. Decalcification with 0.5 M EDTA was performed for cochleae from P7 and older mice. For cryosectioning, tissues were equilibrated with a series of ascending concentrations of sucrose [10%–30% (w/v) in PBS] and then treated serially with a mixture of sucrose and optimum cutting temperature (OCT) compound (Sakura Finetek; 1:1, 3:7, 9:1, then 0:1) in a vacuum chamber for 1 h at room temperature. Tissues were then sectioned (10 μm thick) and processed for immunostaining.

### Sphere-Forming Assay and Differentiation Assay

Fzd9^CreER/+^Rosa26-tdTomato mice were I.P. injected with tamoxifen (4 mg/25 g body weight, Sigma) at P3 and killed at P5, and the cochleae were dissected and digested with trypsin into single cells for FAC sorting of Fzd9+ cells. The cochleae of P5 Lgr5-EGFP^CreER/+^ mice were also dissected and digested with trypsin into single cells for FAC sorting of Lgr5+ cells. For FAC sorting, we used 2 or 3 litters of P5 mice (usually 15–30 mice) to sort the Lgr5+ and Fzd9+ cells (Supplementary Table S1). The sorted Fzd9+ and Lgr5+ cells were cultured in DMEM/F12 medium supplemented with N2 (1:100 dilution, Invitrogen), B27 (1:50 dilution, Invitrogen), heparin sulfate (50 ng/ml, Sigma), and the growth factors bFGF (10 ng/ml, Sigma), EGF (20 ng/ml, Sigma), and IGF-1 (50 ng/ml, Sigma). For the sphere-forming assay, the cells were cultured at a density of 2 cells/μl (200 cells per well) in Costar ultra-low attachment dishes for 5 days. Sphere number and the mean diameter of all the spheres in each well were quantified at the end of the culture. For the differentiation assay, cells were cultured at a density of 20 cells/μl (2,000 cells per well) in a 4-well dish for 10 days. EdU (10 μM, Invitrogen) was added to the culture medium from day 4 to day 7 to label proliferating cells. Immunofluorescence staining was performed at the end of the culture to quantify Myo7a+ and EdU+ cells. The Click-it EdU imaging kit (Invitrogen) was used after blocking to label proliferating cells. Consistent with previous reports (Chai et al., 2012), cell cluster with more than five cells was identified as a sphere or colony. The Myo7a+ cells inside of the colony were considered mitotically generated Myo7a+ cells, while the Myo7a+ cells outside of the colony were considered to be directly differentiated Myo7a+ cells.

### RNA Extraction and RT- PCR

Approximately 20 cochleae from wild-type mice at different ages were used to extract total RNA with Trizol (Thermo). RNA was reverse transcribed into cDNA, and RT-PCR was used to quantify the gene expression levels with *β-actin* as the reference endogenous gene. The primers were as follows: *β-actin*: (F) 5′-ACG GCC AGG TCA TCA CTA TTG-3′; (R) 5′-AGG GGC CGG ACT CAT CGT A-3′; *Fzd9*: (F) 5′-CGC ACG CAC TCT GTA TGG AG-3′; (R) 5′-GCC GAG ACC AGA ACA CCT C-3′.

### Statistical Analysis

For each experimental condition, at least three independent experiments were performed. Data were analyzed with GraphPad Prism6 software and presented as means ± standard errors of the means. Two-way ANOVA was used to determine the statistical significance, and a value of *p* < 0.05 was considered statistically significant. Error bars and *n* values are defined in the respective figures and legends.

## Results

### Lineage Labeling of Fzd9+ Cells in the Neonatal Cochlea From Fzd9-CreER/Rosa26-tdTomato Mice

**Figure 1:**
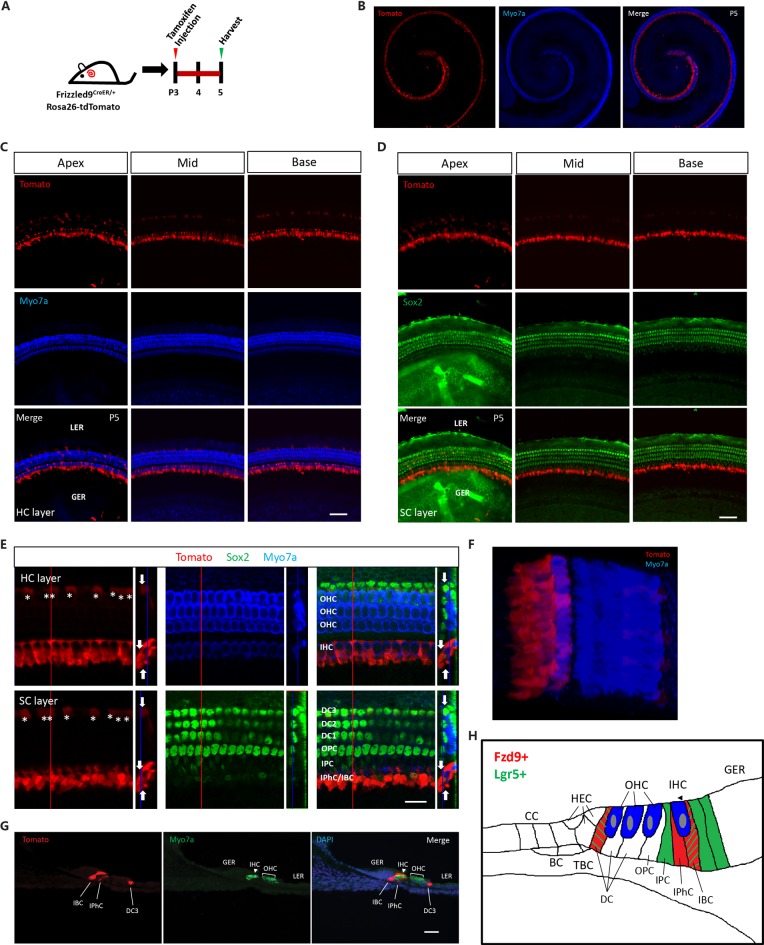
The expression of Fzd9 in the neonatal mouse cochlea. **(A)** Tamoxifen was I.P. injected into P3 Fzd9-CreER/Rosa26-tdTomato mice to activate cre, and the cochleae were dissected 48 h later to identify the tdTomato expression pattern, which indicates Fzd9 expression. **(B–G)** Fzd9 expression was shown by tdTomato. The low-magnification images **(B)** showed that Fzd9 is expressed in the cochlea. The HC layer **(C)** and SC layer **(D)** showed that Fzd9 is expressed in the SCs, but not in the HCs. The high-magnification figures showed that Fzd9 is expressed in IPhCs, IBCs, and the third-row DCs (indicated by white *; **E**). The large square images are single XY slices, the vertical red line shows the position of the orthogonal slice that is shown to the right of each panel, and the blue line on the orthogonal slice shows the level of the XY slice to the left. Orthogonal projections of tdTomato+ cells are indicated by white arrows. A three-dimensional reconstruction of Fzd9 expression is shown in **(F)**. The cryosections also showed the same Fzd9 expression pattern **(G)**. Myo7a was used as the HC marker and Sox2 was used as the SC marker. Scale bar, 50 μm in **(C,D,G)** and 20 μm in **(E)**. **(H)** Schematic of Fzd9 expression in the neonatal mouse cochlea. HC, hair cell; SC, supporting cell; IHC, inner hair cell; OHC, outer hair cell; DC, Deiters’ cell; OPC, outer pillar cell; IPC, inner pillar cell; IPhC, inner phalangeal cell; IBC, inner border cell; GER, the lateral greater epithelial ridge; LER, lesser epithelial ridge; TBC, tympanic border cells; CC, Claudius cells; HEC, Hensen’s cells; BC, Boettcher cells.

We used Fzd9-CreER/Rosa26-tdTomato double-positive mice to lineage label the Fzd9+ cells. Tamoxifen was I.P. injected into P3 Fzd9-CreER/Rosa26-tdTomato double-positive mice to activate cre, and thus Fzd9+ cells were labeled by tdTomato fluorescence after 48 h (Figure 1A). tdTomato-labeled Fzd9+ cells were observed from the apex to the base in the cochlea (Figure 1B), and tdTomato-labeled Fzd9+ cells were found in the SC layer (Figures 1C,D), including the IPhCs and IBCs and to a lesser extent the third-row DCs as shown by the high-resolution images (Figures 1E,F, Supplementary Figure S2). The cryosections showed the same results (Figure 1G). We also compared the lineage labeling of Lgr5+ and Fzd9+ cells in the neonatal mouse cochlea (Figure 1H). Lgr5+ cells have been reported to include the IPCs, IBCs, the third-row DCs, and part of the GER cells (Chai et al., 2011, 2012; Shi et al., 2012), while tdTomato-labeled Fzd9+ cells included the IPhCs, IBCs, and the third-row DCs, but did not include IPCs or GER cells. The cell types and number of Fzd9+ cells were much lower than Lgr5+ progenitors, which suggests that Fzd9+ cells might be a subset of HC progenitors.

### The Lineage Labeling Efficiency of Cochlear Fzd9+ Cells Decreased With Age

**Figure 2:**
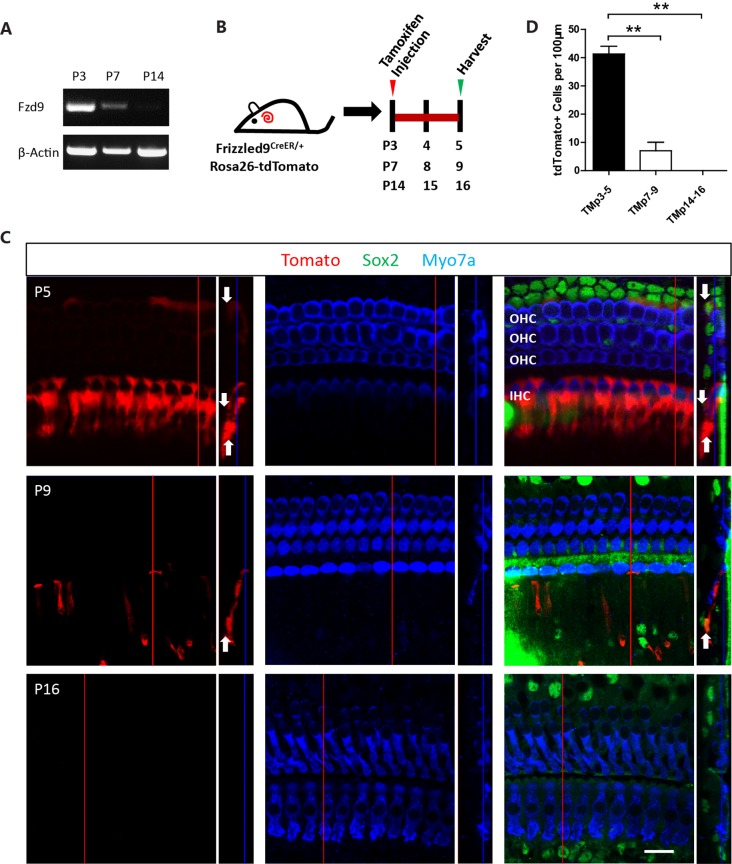
The spatiotemporal expression of Fzd9 in the cochlea at different time points. **(A)** RT-PCR showed *Fzd9* mRNA expression at P3, P7, and P14. *β-actin* was used as the internal control. **(B)** Tamoxifen was I.P. injected into P3, P7, and P14 Fzd9-CreER/Rosa26-tdTomato mice to activate cre, and the cochleae were dissected 48 h later to determine the tdTomato expression pattern, which shows Fzd9 expression at different time points **(C)**. Myo7a was used as the HC marker, and Sox2 was used as the SC marker. The large square images are single XY slices, the vertical red line shows the position of the orthogonal slice that is shown to the right of each panel, and the blue line on the orthogonal slice shows the level of the XY slice to the left. Orthogonal projections of tdTomato+ cells are indicated by white arrows. Scale bar, 20 μm. **(D)** Quantification of tdTomato+ cells at each time point. *n* = 3 for both Lgr5 and Fzd9 mice. **p < 0.01.

The expression of Lgr5 decreases as the mice age and is only expressed in the third-row DCs in the adult mouse cochlea (Chai et al., 2011). Thus we speculated that the lineage labeling efficiency of cochlear Fzd9+ cells might also decrease with age, and we examined the lineage labeling efficiency of cochlear Fzd9+ cells at different time points. First, we measured the mRNA level of *Fzd9* by RT-PCR and found that the *Fzd9* mRNA level decreased dramatically with age and that very little *Fzd9* mRNA expression could be detected at P14 (Figure 2A). Tamoxifen was I.P. injected into P3, P7, and P14 mice, and the mice were killed 48 h later to examine the lineage labeling efficiency of cochlear Fzd9+ cells (Figure 2B). The results showed that consistent with the *Fzd9* mRNA expression level, tdTomato-labeled Fzd9+ cells were dramatically decreased with age, and no tdTomato+ cells could be detected in P14 mice (Figures 2C,D).

### Fzd9+ Cells Generated Similar Numbers of New HCs Compared to Lgr5+ Progenitors in the Neonatal Mouse Cochlea *in vivo*

**Figure 3:**
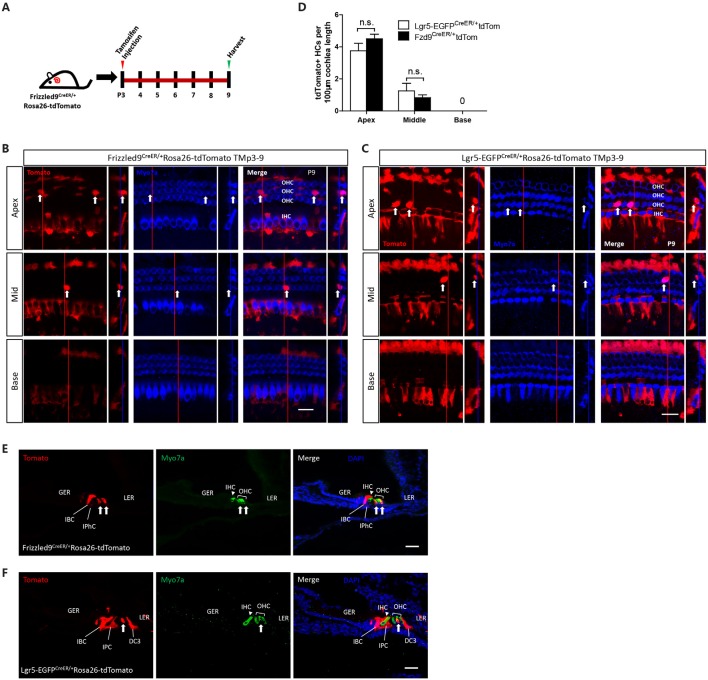
Lineage tracing of Fzd9+ cells in the neonatal mouse cochlea. **(A)** Tamoxifen was I.P. injected into P3 Fzd9-CreER/Rosa26-tdTomato mice to activate cre, and the cochleae were dissected at P9 to lineage trace Fzd9+ cells. Lgr5-EGFP-CreER/Rosa26-tdTomato mice were treated by the same way to lineage trace Lgr5+ cells. **(B–F)** Lineage tracing results showed tdTomato+/Myo7a+ HCs (indicated by white arrows) in the apical, middle, and basal turns of the cochlea by whole-mount **(B,C)** and cryosection **(E,F)**. The large square images are single XY slices, the vertical red line shows the position of the orthogonal slice that is shown to the right of each panel, and the blue line on the orthogonal slice shows the level of the XY slice to the left. Myo7a was used as the HC marker, and Sox2 was used as the SC marker. Scale bar, 20 μm in **(B,C)** and 50 μm in **(E,F)**. Quantification of tdTomato+ HCs was shown in **(D)**. *n* = 4 for Lgr5 mice and *n* = 3 for Fzd9 mice. n.s., not significant.

Next, we used lineage tracing to determine the HC generation ability of Fzd9+ cells *in vivo*. Tamoxifen was I.P. injected at P3 into Fzd9-CreER/Rosa26-tdTomato and Lgr5-EGFP-CreERT2/Rosa26-tdTomato mice to lineage trace the Fzd9+ and Lgr5+ cells, respectively, and the mice were killed at P9 to detect the newly generated tdTomato+/Myo7a+ HCs (Figure 3A). In both Fzd9-CreER/Rosa26-tdTomato and Lgr5-EGFP-CreERT2/Rosa26-tdTomato mice, tdTomato+/Myo7a+ HCs could be found in the apical and middle turns of P9 cochleae (Figures 3B,C), and Fzd9+ cells could generate similar numbers of new HCs compared to Lgr5+ progenitors (Figure 3D). Cryosection images showed the same results (Figures 3E,F). tdTomato-labeled Fzd9+ cells included the IPhCs, IBCs, and third-row DCs, and these Fzd9+ cells could generate similar numbers of new HCs compared to Lgr5+ progenitors, which included more cell types, in neonatal mice cochleae *in vivo*, which suggests that Fzd9+ cells might be HC progenitors.

### Flow Cytometry-Isolated Fzd9+ Cells Have Similar Sphere-Forming Ability Compared to Lgr5+ Progenitors *in vitro*

**Figure 4:**
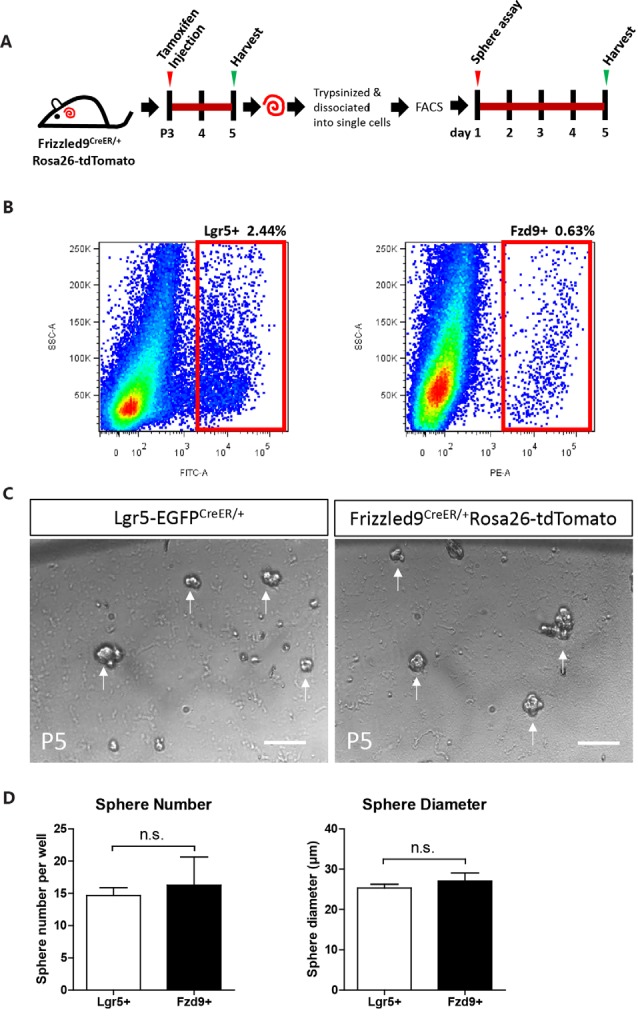
Fzd9+ cells form spheres similarly to Lgr5+ progenitors when cultured *in vitro*. **(A,B)** Tamoxifen was I.P. injected into P3 Fzd9-CreER/Rosa26-tdTomato mice to activate cre, and the cochleae were trypsinized and dissociated into single cells 48 h later for FAC sorting of Fzd9+ cells by tdTomato fluorescence. The cochleae of P5 Lgr5-EGFP-CreER mice were also trypsinized for FAC sorting for Lgr5+ cells. FAC sorting plots are shown in **(B)**. Then Fzd9+ and Lgr5+ cells were cultured *in vitro* for 5 days to form spheres. **(C)** The spheres formed by Lgr5+ progenitors and Fzd9+ cells. Scale bar, 50 μm. **(D)** Quantification of the sphere diameter and number of Lgr5+ spheres and Fzd9+ spheres. *n* = 3 for Lgr5+ spheres and *n* = 4 for Fzd9+ spheres. n.s., not significant.

One of the important characteristics of stem cells and progenitor cells is the ability to self-renew. Fzd9+ and Lgr5+ cells were isolated from Fzd9-CreER/Rosa26-tdTomato and Lgr5-EGFP-CreERT2 mice, respectively, by flow cytometry, and the cells were cultured *in vitro* for 5 days to form spheres (Figure 4A). The flow cytometry plots showed that the Lgr5+ progenitors were around 2.44% of the whole cochlear cell population, while the Fzd9+ cells were only around 0.63% of the whole cochlear cell population (Figure 4B). The sphere-forming assay showed that 200 Fzd9+ cells could form a similar number of spheres compared to 200 Lgr5+ progenitors (Figures 4C,D, Supplementary Figure S3), and the statistical analysis showed that the diameters of the spheres generated from Fzd9+ cells and Lgr5+ progenitors were almost the same (Figure 4D and Supplementary Table S1). Together, these results suggested that Fzd9+ cells had almost the same proliferation and sphere-forming ability as Lgr5+ progenitors.

### Flow Cytometry-Isolated Fzd9+ Cells Have Similar Differentiation Ability for Generating Myo7a+ Cells Compared to Lgr5+ Progenitors *in vitro*

**Figure 5:**
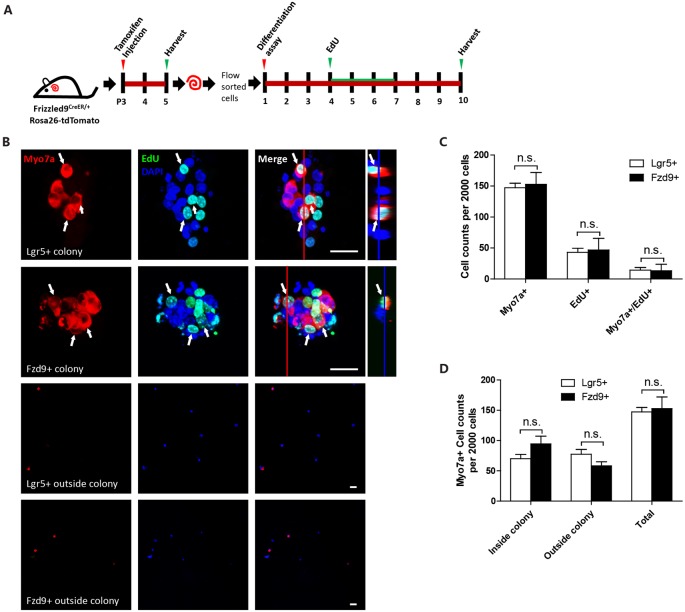
Sphere differentiation assay of Fzd9+ and Lgr5+ cells. **(A)** The same numbers of Fzd9+ cells and Lgr5+ progenitors were cultured *in vitro* for 10 days and allowed to differentiate. **(B)** Immunofluorescence images of differentiated spheres and individual cells outside the colonies formed from Fzd9+ cells and Lgr5+ progenitors. The large square images are single XY slices, the vertical red line shows the position of the orthogonal slice that is shown to the right of each panel, and the blue line on the orthogonal slice shows the level of the XY slice to the left. Myo7a was used as the HC marker, and EdU was used to label proliferating cells. Myo7a+/EdU+ cells are indicated by white arrows. Scale bar, 20 μm. **(C,D)** Quantification of Myo7a+ and EdU+ cells in spheres **(C)** and Myo7a+ cells in inside and outside colonies **(D)** formed from Fzd9+ cells and Lgr5+ progenitors. *n* = 3 for both Lgr5+ and Fzd9+ cells. n.s., not significant.

Next, we examined the differentiation ability of isolated Fzd9+ cells. We isolated the Fzd9+ and Lgr5+ cells, respectively, by flow cytometry and then performed a differentiation assay to culture the cells *in vitro* for 10 days, and EdU was added from day 4 to day 7 to detect the proliferating cells (Figure 5A). We found that 2,000 Fzd9+ cells generated similar numbers of Myo7a+ cells compared to 2,000 Lgr5+ progenitors after 10 days of differentiation and that 2,000 Fzd9+ cells also generated similar numbers of Myo7a+/EdU+ cells compared to 2,000 Lgr5+ progenitors (Figures 5B–D, Supplementary Table S1). Together this suggested that Fzd9+ cells have similar differentiation ability for generating Myo7a+ cells compared to Lgr5+ progenitors.

## Discussion

The loss of HCs is the main cause of sensorial hearing loss, and HC regeneration from HC progenitors has become one the most important areas of research in the field of hearing research. Lgr5 has been reported to be a marker of HC progenitors that can regenerate HCs in the neonatal mouse cochlea. However, Lgr5+ cells can be found among many cell types and in different numbers of cells. Here, we show that Fzd9 can be used as a more restricted HC progenitor marker that only marks IPhCs, IBCs, and third-row DCs. We found that Fzd9+ cells can generate HCs in the neonatal mouse cochlea *in vivo* and can form spheres and generate HCs when cultured *in vitro*.

Fzd9, one of the 10 frizzled family proteins, acts as a Wnt receptor and can activate canonical and non-canonical Wnt signaling pathways (Karasawa et al., 2002; Winn et al., 2005; Avasarala et al., 2013a,b; Heilmann et al., 2013; Aviles et al., 2014), and has been reported to interact with Wnt2, Wnt5, Wnt7, and Wnt8 to activate different downstream signaling cascades (Karasawa et al., 2002; Momoi et al., 2003; Winn et al., 2005; Avasarala et al., 2013a,b; Ramírez et al., 2016). Further research is needed to determine which Wnt proteins Fzd9 interacts within the cochlea and whether it activates the canonical or non-canonical pathway. In the brain and 293T cell line, upregulation of Fzd9 leads to the induction of Wnt signaling, while downregulation of Fzd9 inhibits Wnt signaling (Karasawa et al., 2002; Ramírez et al., 2016). In the inner ear, many previous reports, including our own findings, have shown that activation of Wnt signaling significantly promotes the proliferation of Lgr5+ progenitors both *in vitro* and *in vivo* and that inhibition of Wnt signaling significantly decreases the proliferation ability of Lgr5+ cells (Chai et al., 2012; Shi et al., 2012; Li et al., 2015; Ni et al., 2016; Wu et al., 2016). Furthermore, in 3D cultures of Lgr5+ progenitors, activation of Wnt signaling increases the formation of 3D organoids (Lenz et al., 2019). Thus it is possible that Fzd9 might play a role in regulating Wnt signaling in the inner ear, which in turns affects the proliferation ability of Fzd9+/ Lgr5+ progenitors. And the detailed role of Fzd9 in regulating the proliferation and/or differentiation of the inner ear progenitors still need to be investigated in the future. Many other genes and signaling pathways, such as Notch, Neurog1, Hedgehog, and Foxg1, have been reported to play a role in HC development and regeneration (Pauley et al., 2006; Jahan et al., 2015a,b; Li et al., 2015; Ni et al., 2016; Chen et al., 2017; He et al., 2019), and we will study their roles in Fzd9+ progenitors in the future.

Lgr5+ progenitors have been shown to include IPCs, IBCs, third-row DCs, and some of the GER cells, while Fzd9+ cells include IPhCs, IBCs, and third-row DCs (Figure 1H), and we used Sox2 to label SCs (Figures 1D,E, Supplementary Figure S2), including Hensen’s cells (HECs), DCs, outer pillar cells (OPCs), IPCs, IPhCs, IBCs and GER (Dabdoub et al., 2008; Nichols et al., 2008; Chai et al., 2012; Li et al., 2015). In the very apical turn of the cochlea, only 4.8 ± 1.7 OPCs, first-row and second-row DCs per 100 μm of the cochlea length were labeled by tdTomato, while we did not find any tdTomato+ OPCs, first-row or second-row DCs in the rest of the apical, middle, and basal turns. The flow cytometry plots also showed that Lgr5+ progenitors made up around 2.44% of all of the cochlear cells, while the Fzd9+ cells only made up 0.63% of the total cochlear cells (Figure 4B). Thus the cell types and cell numbers of Fzd9+ cells were both much less than Lgr5+ cells, which means that Fzd9+ cells were a smaller cell population compared to Lgr5+ cells. Considering that the proliferation, differentiation, and HC generation ability of Fzd9+ cells is similar to Lgr5+ progenitors, it is very possible that Fzd9+ cells are the main functional progenitor cell types among Lgr5+ cells, and thus our work provides a new marker for HC progenitors and narrows down the progenitor cell types and numbers compared to Lgr5+ progenitors. According to our current data, we speculated that Lgr5+/Fzd9- SCs might have very limited ability as HC progenitors, which still need further more research to prove. IBCs and the third-row DCs express both Lgr5 and Fzd9, while IPhCs only express Fzd9, and this suggests that IPhCs might also be important for HC generation. Our recent research reported that Lgr6 is expressed in the IPCs, which have higher ability for differentiation and lower ability for proliferation compared to Lgr5+ progenitors (Zhang et al., 2018). Fzd9+ IPhCs express neither Lgr5 nor Lgr6, but these cells have never been studied in terms of their ability to proliferate and differentiate. In a future study, we are planning on flow sorting the Fzd9+ IPhCs to determine their proliferation and differentiation ability.

Fzd9 expression decreased dramatically with age, which is consistent with the decreased expression of Wnt signaling with age (Chai et al., 2011; Jan et al., 2013). Fzd9 is expressed at high levels at P3, at much lower levels at P7, and is undetectable at P14, which is consistent with the rapid decrease of HC generation ability in the mouse cochlea after P7. These results also suggested that Fzd9 could serve as a marker for HC progenitors because its expression correlates with the HC generation ability of the postnatal mouse cochlea.

The sphere-forming assay showed that Fzd9+ cells could form spheres when cultured *in vitro* and that these spheres were similar in number and diameter compared to Lgr5+ progenitors. We also performed a sphere-forming assay in older mice to see if the subpopulation of Lgr5+/Fzd9+ cells loses their proliferative capacity differently. P9 Fzd9-CreER/Rosa26-tdTomato mice were injected with tamoxifen, and Fzd9+ cells were FAC sorted at P12 (Supplementary Figure S1A). P12 Lgr5-EGFP-CreER mice were also used for FAC sorting Lgr5+ progenitors. Both cells were cultured *in vitro* for the sphere-forming assay, and we observed fewer and smaller spheres at P12 than at P5, and the sphere numbers and mean sphere diameters generated from P12 Lgr5+ cells and Fzd9+ cells were similar after 5 days of cell culture (Supplementary Figures S1B,C), which was consistent with previous reports that showed the sphere-forming ability were decreasing with age (Oshima et al., 2007). This suggests that Fzd9+ cells can proliferate and renew themselves when cultured *in vitro* and that the *in vitro* proliferation ability of Fzd9+ cells and Lgr5+ cells are similar.

Differentiation results showed that Fzd9+ cells could proliferate and differentiate into Myo7a+ cells, which is consistent with our *in vivo* data. We also compared the differentiation ability of Fzd9+ cells and Lgr5+ cells and found that the numbers of Myo7a+ cells and Myo7a+/EdU+ cells were almost the same, which means that the differentiation ability of Fzd9+ cells and Lgr5+ are practically the same. Taken together, the sphere-forming assay and *in vitro* differentiation assay both showed that when cultured *in vitro* the proliferation and differentiation ability of Fzd9+ cells are similar to Lgr5+ cells and that Fzd9+ cells have similar characteristics as previously identified HC progenitors. One recent report showed that Lgr5+ progenitors could be established as a valuable *in vitro* tool for the analysis of progenitor cell manipulation and HC differentiation (Lenz et al., 2019), and this has inspired us to establish Fzd9+ progenitors for the same use in the future.

In summary, we used Fzd9-CreER/Rosa26-tdTomato mice to show that Fzd9+ cells were traced in IPhCs, IBCs, and third-row DCs, but not in IPCs or GER cells, in the neonatal mouse cochlea and that Fzd9 expression decreased with age. Fzd9+ cells have the ability to generate HCs *in vivo* and form spheres *in vitro*, and the differentiation ability of Fzd9+ cells was similar compared to Lgr5+ progenitors in the *in vitro* differentiation assay. Our work thus suggests that Fzd9 is a more restricted marker for HC progenitors than Lgr5.

## Data Availability

All datasets generated for this study are included in the manuscript and/or the Supplementary Files.

## Ethics Statement

We performed all animal procedures according to protocols that were approved by the Animal Care and Use Committee of Southeast University and that were consistent with the National Institute of Health’s Guide for the Care and Use of Laboratory Animals. We made all efforts to minimize the number of animals used and to prevent their suffering.

## Author Contributions

SZ, XC, XQ, and RC conceived and designed the experiments. SZ, DL, ZZ, YZ, YD, HZ, LG, JQ, and RQ performed the experiments. SZ, MT, XG, CZ, XC, XQ, and RC analyzed the data. SZ, RC, XQ, and XC wrote the article. All authors read and approved the final manuscript.

## Conflict of Interest Statement

The authors declare that the research was conducted in the absence of any commercial or financial relationships that could be construed as a potential conflict of interest.
