# Supplementary material for: Frizzled-9+ Supporting Cells Are Progenitors for the Generation of Hair Cells in the Postnatal Mouse Cochlea
Source: Front Mol Neurosci. 2019 Jul 31;12:184. doi: 10.3389/fnmol.2019.00184 (PMC6689982; doi:10.3389/fnmol.2019.00184)
Supplement: Supplementary file 1 [file Data_Sheet_1.docx]

**Supplementary Information**

**Supplementary Table 1**

**Information of FAC sorting and cell culture**

| Mice genotype | Fzd9-CreER/Rosa26-tdTomato | Lgr5-EGFP-CreER |
| --- | --- | --- |
| Tamoxifen injection | P3 - P5 | None |
| Number of mice used for FAC sorting | 2 or 3 litters (usually 15–30 P5 or P12 mice) | |
| FAC sorted cell number | 2k-10k tdTomato+ cells | 8k-40k EGFP+ cells |
| Cell number for sphere assay | 200 cells per well (2 cells/μl) | |
| Sphere number (P5) | 16.3 ± 4.4 | 14.7 ± 1.2 |
| Sphere diameter (P5) (μm) | 27.1 ± 2.0 | 25.3 ± 0.9 |
| Sphere number (P12) | 6.7 ± 0.7 | 7.3 ± 1.2 |
| Sphere diameter (P12) (μm) | 22.8 ± 1.1 | 22.2 ± 1.2 |
| Cell number for differentiation assay | 2000 cells per well (20 cells/μl) | |
| Myo7a+ cells per well | 152.7 ± 19.23 | 147.3 ± 7.2 |
| EdU+ cells per well | 46.7 ±19.0 | 43.0 ± 6.5 |
| Myo7a+/EdU+ cells per well | 13.3 ± 10.5 | 14.3 ± 4.3 |
| Myo7a+ cells of inside colony per well | 94.3 ± 12.9 | 70.0 ± 7.0 |
| Myo7a+ cells of outside colony per well | 58.3 ± 6.6 | 77.3 ± 7.8 |

**Supplementary Figure Legends**

**Figure S1.** P12 Fzd9+ cells form spheres similarly to Lgr5+ progenitors when cultured in vitro. (A) Tamoxifen was I.P. injected into P9 Fzd9-CreER/Rosa26-tdTomato mice to activate cre, and the cochleae were trypsinized and dissociated into single cells at P12 for FAC sorting of Fzd9+ cells by tdTomato fluorescence. The cochleae of P12 Lgr5-EGFP-CreER mice were also trypsinized for FAC sorting for Lgr5+ cells. Then Fzd9+ and Lgr5+ cells were cultured in vitro for 5 days to form spheres. (B) The spheres formed by Lgr5+ progenitors and Fzd9+ cells. Scale bar, 50 µm. (C) Quantification of the sphere diameter and number of Lgr5+ spheres and Fzd9+ spheres. n = 3 for both Lgr5+ spheres and Fzd9+ spheres. n.s., not significant.

**Figure S2.** Z-projection of Sox2 staining of the cochlea. DC, Deiters’ cell; OPC, outer pillar cell; IPC, inner pillar cell; IPhC, inner phalangeal cell; IBC, inner border cell; GER, the lateral greater epithelial ridge; LER, lesser epithelial ridge; HEC, Hensen’s cells. Scale bar, 50 µm.

**Figure S3.** High-resolution images of spheres formed by Fzd9+ and Lgr5+ cells. Tamoxifen was I.P. injected into P3 Fzd9-CreER/Rosa26-tdTomato mice to activate cre, and the cochleae were trypsinized and dissociated into single cells 48 h later for FAC sorting of Fzd9+ cells by tdTomato fluorescence. The cochleae of P5 Lgr5-EGFP-CreER mice were also trypsinized for FAC sorting for Lgr5+ cells. Then Fzd9+ and Lgr5+ cells were cultured *in vitro* for 5 days to form spheres and then DAPI was used to stain the nucleus. Scale bar, 20 µm.
