# Supplementary figures and images for: Frizzled-9+ Supporting Cells Are Progenitors for the Generation of Hair Cells in the Postnatal Mouse Cochlea
Source: Front Mol Neurosci. 2019 Jul 31;12:184. doi: 10.3389/fnmol.2019.00184 (PMC6689982; doi:10.3389/fnmol.2019.00184)

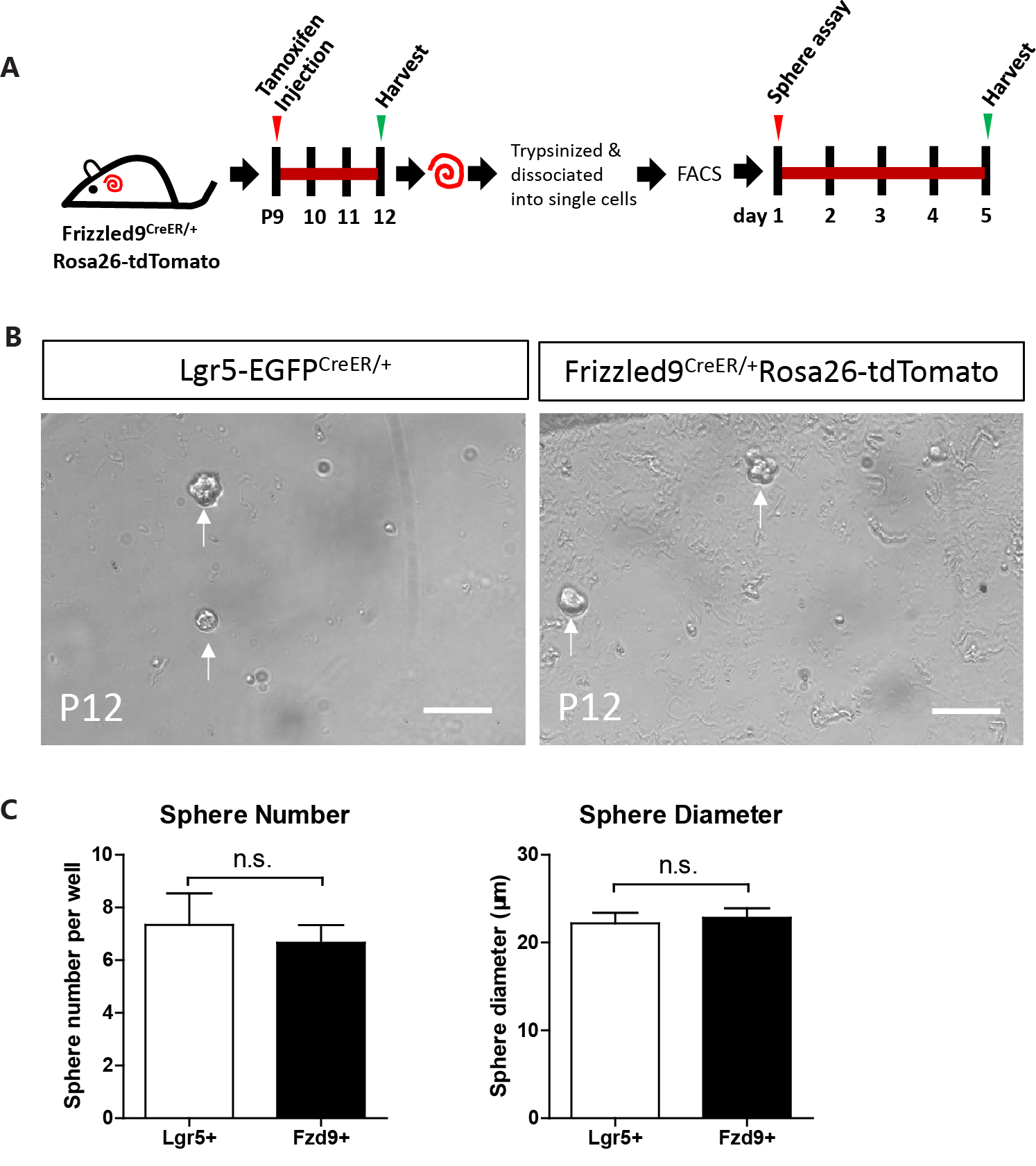

Supplement: Supplementary file 2 [file Image_1.TIF]

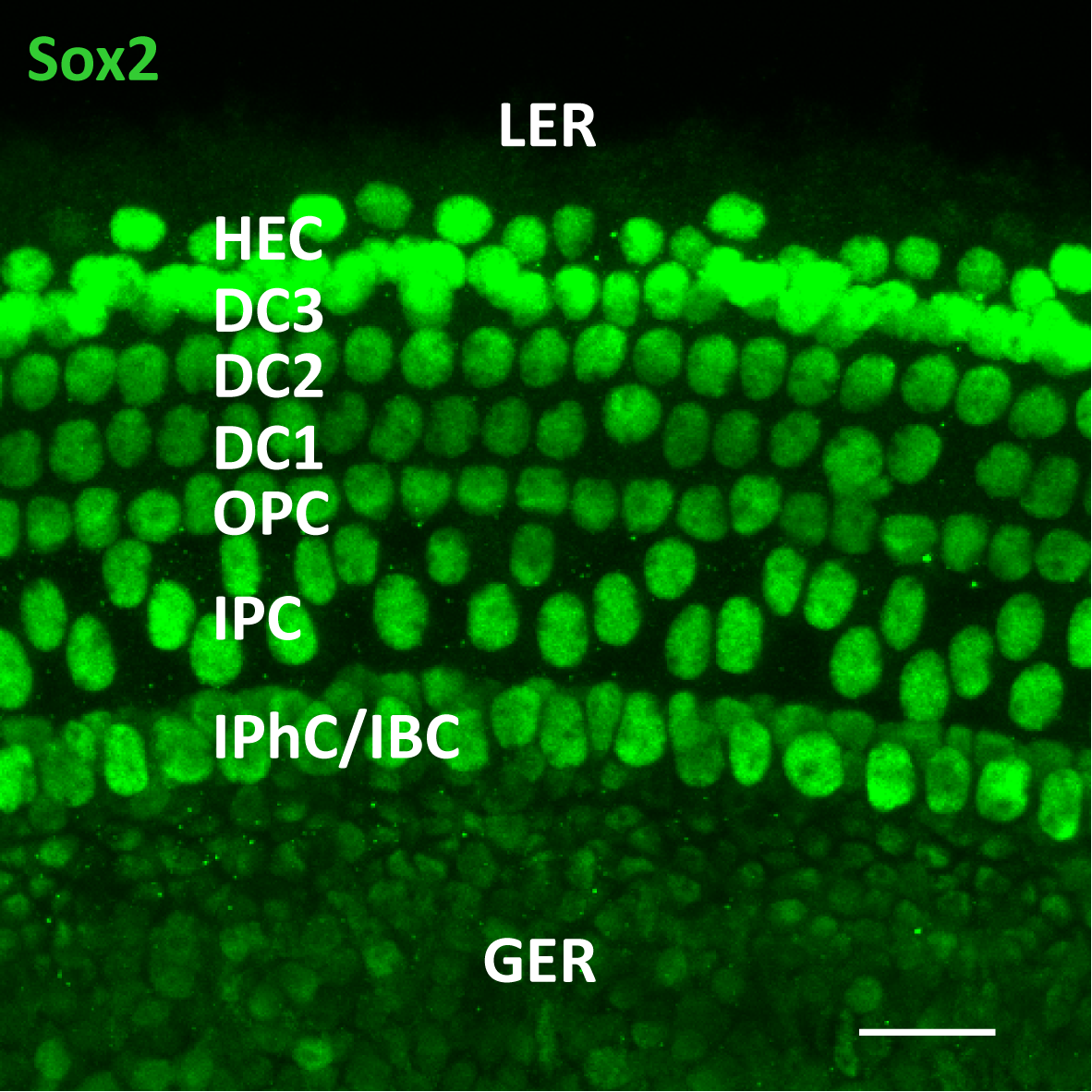

Supplement: Supplementary file 3 [file Image_2.TIF]

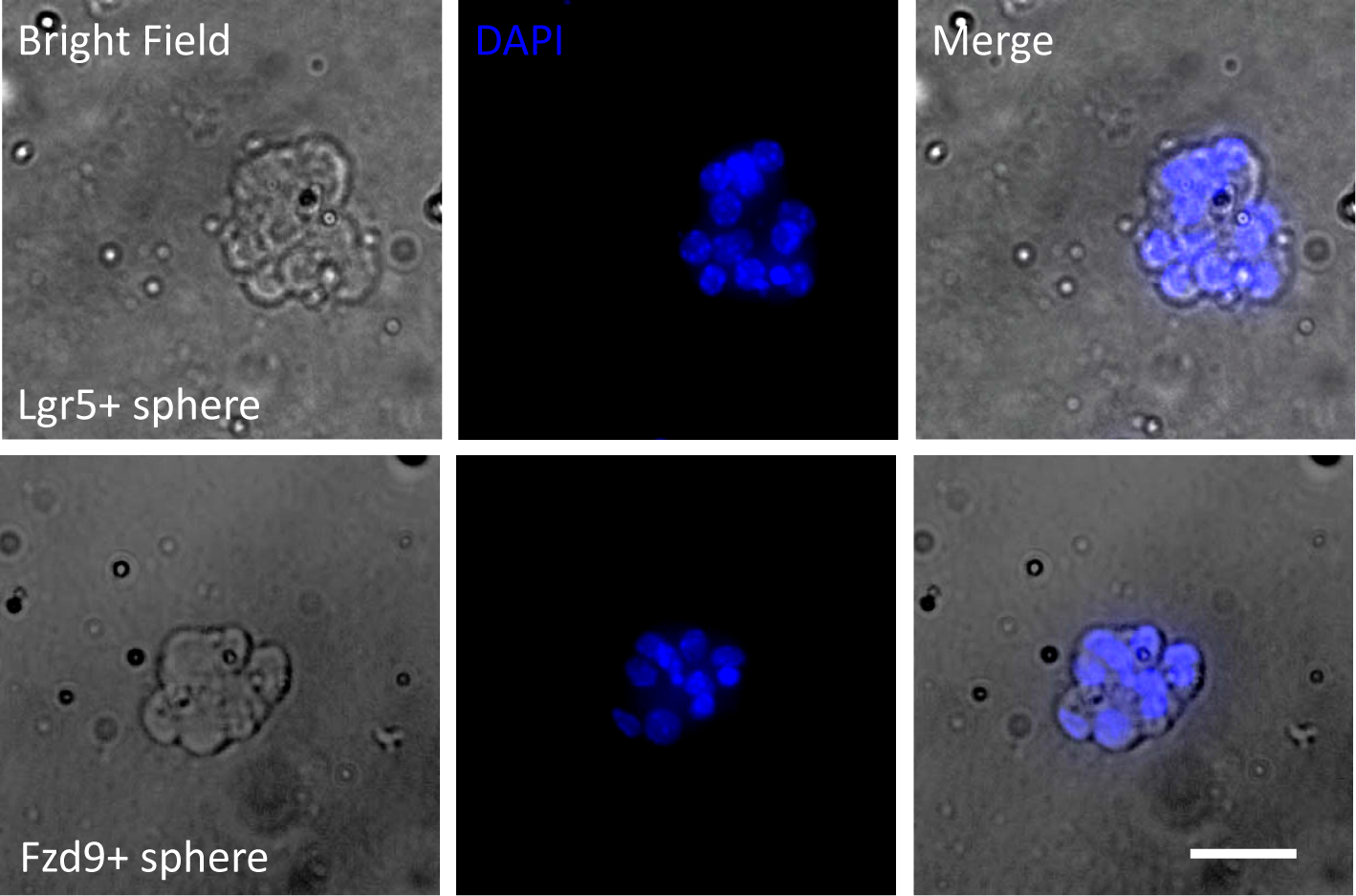

Supplement: Supplementary file 4 [file Image_3.TIF]
